# Healthy young adult relationships (HYAR): professionals' perspectives on HYAR education and training

**DOI:** 10.1080/02673843.2026.2670387

**Published:** 2026-05-08

**Authors:** Niamh Hurst, Jessica Hassan, Susan Lagdon, Lucia Klencakova, Ciaran Shannon, Cherie Armour, Mark Tully, Paul Best, Raymond Bond, Aine Aventin, Julie-Ann Jordan

**Affiliations:** a Impact Research Centre, Northern Health and Social Care Trust, Antrim, Northern Ireland, United Kingdom; b School of Psychology, Ulster University, Coleraine, Northern Ireland, United Kingdom; c School of Psychology, Queen's University Belfast, Belfast, Northern Ireland, United Kingdom; d School of Medicine, Ulster University, Derry-Londonderry, Northern Ireland, United Kingdom; e School of Social Sciences, Education and Social Work, Queen's University Belfast, Belfast, Northern Ireland, United Kingdom; f School of Computing, Ulster University, Belfast, Northern Ireland, United Kingdom; g School of Nursing and Midwifery, Queen's University Belfast, Belfast, Northern Ireland, United Kingdom

**Keywords:** Coercive control, healthy young adult relationships, youth work, relationships and sexuality education, intervention, health and wellbeing

## Abstract

The onus for educating and supporting young people in navigating romantic relationships often falls on professional youth work organizations; yet, evidence relating to healthy young adult relationship (HYAR) education programmes delivered within these settings remains limited. The present study sought to capture youth work professionals' views as part of a research programme focused on developing HYAR education resources for young people. Eleven youth work professionals in Northern Ireland participated in individual interviews or focus groups (5 interviews and 2 focus groups) between September and November 2024. Three master themes were identified using Reflexive Thematic Analysis: (1) defining and understanding coercive control; (2) barriers to communicating and reporting; and 3) training needs. The findings highlight professionals' perceptions of HYAR education and training needs of young people, parents and professionals. Participating professionals believed that youth work organizations should assume a key role in delivering HYAR education in a meaningful and accessible way.

## Introduction

Forming and maintaining romantic relationships is often considered a central developmental feature of adolescence and young adulthood (Shulman & Connolly, 2013), and experiences arising from these relationships can have a lasting impact (Exner-Cortens et al., 2013; Piolanti et al., 2023). How ‘healthy’ relationships are defined, described, and measured during this period varies considerably across the literature, yet there is a consistent focus placed on the presence (or absence) of certain relationship characteristics and behaviours, such as caregiving, respect, and support (Benham-Clarke et al., 2022; Cavaletto et al., 2025). Many young people may experience unhealthy relationships in the form of coercive control, which occurs in relationships when one partner seeks to assert and maintain dominance over the other partner (Pitman, 2017; Stark, 2007). This may take the form of making credible threats of violence or imposing restrictions on a person's freedom and independence. Further, certain coercive control tactics can be particularly salient amongst young people, such as ‘love-bombing’ (i.e. attempting to influence an individual through excessive demonstrations of attention and affection) and ‘gaslighting’ (i.e. behaviour that manipulates another person, causing them to doubt their memories, perceptions and beliefs) (Spear, 2019; Martin et al., 2012). Digital and technology-facilitated abuse also appears to be an increasingly prevalent issue among young people (Øverlien et al., 2020).

Limited research has examined how young people themselves perceive potential indicators of healthy and unhealthy relationships. Among the studies that have examined this, many report that young people identify relationship characteristics such as respect, trust, and open communication as being indicative of a healthy relationship (Gowan et al., 2014), whilst abuse, disrespect, monitoring each other's whereabouts or activities, or ‘guilt-tripping,’ one another were viewed unfavourably (Martin et al., 2012). Of concern, young people often normalize unhealthy relationship behaviours, including experiences of intimate partner violence (IPV) and may be more likely to accept and rationalize coercive and/or controlling behaviours specifically (Abbott, Weckesser & Egan, 2021). This could be partially attributed to a lack of understanding regarding coercive control and the associated risks among young people. Indeed, experiences of coercive control and IPV in young adult relationships have been linked to increased risk of further victimization as a teen and in adulthood, adverse mental health outcomes, and high-risk behaviours (Piolanti et al., 2023). Yet previous research conducted by Lagdon et al. (2023) found that only 16% of 16-year-olds in Northern Ireland (NI) had heard of the term coercive control and knew what it meant. Moreover, young people struggled to recognize the risks associated with more covert, subtle indicators of coercive control (Lagdon et al., 2023), which may be present in the earlier stages of a relationship and can be a predictor for escalating violence (Stansfield & Williams, 2021). In addition, research shows that double standards centred on gender differences may place certain young people, such as young males, at increased risk in relation to coercive control victimization, due to limited concern regarding its impact on these individuals (Abbott, Weckesser & Egan, 2021; Lagdon et al., 2023).

Difficulties in identifying unhealthy relationship indicators and associated gaps in young people's understanding of coercive control, as evidenced in previous literature may be, in part, due to a lack of positive relationship models (Kedzior et al., 2024). Both enduring family influences and exposure to social media content that implicitly promotes sexual violence, misogyny, and pro-aggression attitudes can shape young people's understanding of, and behaviour within, relationships (Fosco et al., 2016). Exposure to IPV in the family of origin, for example, may serve as a distinct pathway to increased risk of victimization and/or perpetration among young people by adversely affecting the development of secure attachments in infancy and facilitating further social learning of aggressive relational strategies (Armour & Sleath, 2014). In instances of parental IPV, young people might be directed by the perpetrator to participate in coercive control activities, such as isolation and monitoring (Stark, 2007), therefore instilling the belief that such behaviours are appropriate and acceptable within one's own intimate relationships. Harsh and overprotective parenting can also normalize the use of controlling behaviours in the context of close relationships (Surjadi et al., 2013). As such, it is evident that the cumulative effect of the family environment and its associated impact on young people's developmental processes may partially underpin intergenerational cycles of IPV (Smith-Marek et al., 2015). This effect may then be indirectly exacerbated by additional influences, such as social media (Terrell et al., 2023).

A meta-analysis by McElwain, McGill & Savasuk-Luxton (2017) concluded that youth-focused relationship education appears to be effective in building conflict management skills and addressing faulty relationship beliefs, although evidence regarding healthy relationship attitudes is still lacking. However, another review points to more glaring gaps in the evidence base, and has highlighted that existing studies are not high quality, there is a need to co-create programmes with young people, teachers and professionals, assess their longitudinal impact robustly, and use a core set of validated outcome measures (Benham-Clarke et al., 2023). Statutory Relationships and Sexuality Education (RSE) guidance issued by the United Kingdom (UK) Department for Education (DfE) stipulates that pupils in England should learn about healthy young adult relationships (HYAR), including the characteristics of healthy and unhealthy relationships, and safety in forming and maintaining relationships (Department for Education, 2019). However, many other regions, including NI, lack a standardized, evidence-based RSE curriculum. Whilst conflicting political and religious beliefs, characteristic of the socio-political climate in NI, often present a significant barrier to the implementation of a mandatory RSE curriculum in this region, similar challenges to the delivery of RSE have been identified elsewhere (Ewing et al., 2020; Fox et al., 2014). For example, RSE delivery in South Australia is largely dependent on individual schools' values and needs, resulting in considerable variability in the content delivered to pupils (Ezer et al., 2019). Similar discrepancies have been evidenced in other countries, such as the United States (US), in which several programmes exist, each varying in terms of duration, programme aims, content and delivery methods (Janssens et al., 2020). Yet additional barriers relating to the knowledge, training and resources afforded to professionals tasked with delivering such education present a further obstacle to the delivery of consistent and high-quality RSE (Benham-Clarke et al., 2023). Despite teachers themselves viewing RSE as a specialist topic (Cole, 2018), many undertake RSE teaching with little to no specialist professional development (Martínez et al., 2012), which appears to contribute to a lack of teaching proficiency, comfortability and confidence with teaching topics associated with RSE (Lohan et al., 2023; Walker et al., 2021). Indeed, further qualitative research among professionals highlights the clear need for appropriate experience and subject knowledge, especially regarding previously overlooked areas such as LGBTQ+ relationships (Cumper et al., 2024).

Taken together, it is unsurprising that young people may rely more heavily on personal experiences and observation of other relationship models, such as those presented by family and social media, rather than formal educational programmes to develop their understanding of ‘healthy’ relationships (Kedzior et al., 2024). Moreover, young people with special educational needs and disabilities (SEND), and those who are home-schooled may face additional barriers to accessing adequate RSE (Harding, 2024), which may further exacerbate disparities in young people's understanding of HYAR. As such, it is clear that additional pathways to education are required to address potential inequalities in the accessibility and quality of HYAR education programmes for young people. As youth work aims to prevent personal and social issues affecting young people and promote their personal development (Sonneveld et al., 2021), community-based delivery methods provided by youth focused professionals may offer an important partnership opportunity in meeting the needs of the RSE curriculum within a flexible format. Indeed, external agencies, such as youth organizations and community-based educators, often develop the resources and lesson plans upon which formal educators rely, and are, at present, frequently recruited to deliver RSE within schools (Benham-Clarke et al., 2023; Parish, 2022). As such, it has been suggested that the youth work sector, in part, helps to address gaps in RSE delivery created by school ethos constraints and conservatism (Parish, 2022; Rolston, Schubotz & Simpson, 2005). Yet such practice remains at the discretion of individual schools, thus contributing to further inconsistencies in the content and delivery of RSE within schools (Benham-Clarke et al., 2023). Beyond this, interventions delivered by youth workers within the community are positioned as being voluntary, in that young people choose whether, and how long to participate, and these tend to be based within various informal contexts, such as youth clubs and arts/sports facilities (Batsleer & Davies, 2010; Ritchie & Ord, 2017). However, there is an evidence gap concerning RSE and HYAR educational programmes delivered by youth work organizations at present.

The present study is part of a research programme seeking to develop HYAR education resources for young people that could be delivered through the youth work sector. Views from a range of stakeholders were sought, including youth work professionals themselves. The current study explores professionals’ perspectives on: youth and parental understandings of healthy and unhealthy young adult relationships; challenges associated with educating young people and parents about such relationships; what are the specific relationship education training needs of young people and those who support them (e.g. parents and professional); and how should these needs be addressed. Through qualitative interviews, we identified ways to improve education, support, and prevention efforts for young people experiencing IPV, including coercive control.

## Method

### Design and participants

Invitations to participate in this research were shared with youth organizations across NI. These organizations had been identified by the wider project steering committee which included members working within the voluntary and youth sector with NI. The email invitation was a condensed version of the larger Participant Information Sheet (attached to the email), which provided a detailed outline of the research's purpose, the tasks and requirements for potential participants, the nature of the interview, and the time commitment required for participation. Inclusion criteria for the participants were: (1) over the age of 18; and (2) a youth work professional currently working with young people within community-based settings. Eleven youth sector professionals across seven different organizations in NI participated in individual interviews or focus groups (5 interviews and 2 focus groups). The youth work professionals predominantly reported their gender identity as ‘woman’ (*n* = 9), with the remaining identifying as ‘men’ or ‘non-binary’. They ranged in age from 25–46 years (M = 35 years) and spanned a number of roles within the sector, including programme managers, project coordinators and life skills facilitators (youth workers). All of the organizations in which these professionals were employed provided youth services, with three operating as community-based youth clubs, two functioning as broader community centres, one specifically supporting LGBTQ young people, and one non-profit offering a wide range of services for children and young people.

### Procedure

The study was granted ethical approval by Ulster University's Research Ethics Committee (REC/22/0018, 8th August 2022). It was carried out in accordance with the Declaration of Helsinki and guided by the Research Integrity Framework on Domestic Violence and Abuse (Women’s Aid Federation of England, Women’s Aid Federation of Scotland, Welsh Women’s Aid & Women’s Aid Federation Northern Ireland, 2020). Professionals interested in participating and getting involved with the project were asked to complete a study consent form and email this to the research team (LK as single point of contact). A time and date were then arranged that would work best for them to participate in an interview or focus group discussion. The option of a focus group format was implemented to accommodate multiple professionals within a single organization. Indeed, time to engage in research activity outside of ‘normal’ work commitments can be a challenge within any sector. Two individual interviews took place online via MS Teams, while the remaining interviews were completed in person. Two focus groups were also conducted (*n* = 2; *n* = 4). Across all data collection methods, session durations ranged from 26 min to 1 h, with an average length of 47 min. There were no significant differences in duration between focus groups and individual interviews, aside from two interviews that extended to the full hour. The interview schedule (see Supplementary materials) focused on understanding professional's perspectives of young people's (un)healthy relationship awareness levels and education needs, and how best to deliver healthy relationship education and awareness raising initiatives in the future. A further focus included exploration of the nature and types of training and awareness raising initiatives that youth supporting professionals have received to support them in working with young people, particularly when addressing issues of domestic abuse and unhealthy relationships. Targeted questions and prompts were used to further examine these issues, particularly in relation to coercive control. At the conclusion of each interview, the researcher provided a summary of the key points discussed and invited participants to confirm or clarify the information shared prior to closing the session.

### Analysis

Data analysis was conducted in accordance with the principles of reflexive thematic analysis (Braun & Clarke, 2021). Two researchers (JH & NH) familiarized themselves with the transcripts; specifically, NH was allocated four transcripts at this stage and JH the other three. Each researcher independently identified initial open codes and emerging themes within the transcripts allocated to them, prior to meeting with a third researcher (JAJ) to discuss interpretation and facilitate understanding of the meaning from multiple perspectives. Codes and initial themes were then merged, refined and/or renamed throughout this process, as the two researchers moved backwards and forwards through the six analytical phases, prior to meeting with the third researcher for further discussion. Initial themes were then collaboratively merged and refined. Consensus was reached among the research team (JH, NH, JAJ, SL & LK) on the thematic structure of the data. Data saturation was reached when no new themes, codes, or information were identified from the interviews and team consensus regarding theoretical sufficiency was reached (Braun & Clarke, 2021).

### Positionality statement

As qualitative methods were utilized to address the research questions, the positioning of the researchers in conducting the present study is therefore acknowledged. A critical realist paradigm was adopted, whereby experiences of abuse (including coercive control) in young adult relationships were understood to reflect material and psychological realities (Bhaskar, 1979), whilst recognizing that accounts of these experiences are socially situated and mediated through social, cultural and professional discourses (Fletcher, 2017).

As a regional research team based in NI, all researchers were embedded within the social and cultural context in which the present research was conducted. Two members of the research team (SL & LK) were involved in the research development and data collection. Data analysis was conducted by three members of the team (NH, JH & JAJ), whose interpretations were informed by disciplinary backgrounds in psychology and prior research pertaining to coercive control and IPV more broadly, shaping their analytic lens and theoretical sensitivity to patterns of power and control. Reflexivity strategies including reflexive memoing, team-based analytic discussions, and actively challenging assumptions during coding were employed throughout theme development to enhance transparency and critically examine the interpretative role of the researchers. Interpretative decisions were shaped by the researchers' subjectivities (Braun et al., 2023), including their ongoing work focused on developing a healthy relationships educational app for young people, which may have heightened attentiveness to prevention and early identification in relation to unhealthy relationships.

## Results

Three master themes were identified; namely, (1) defining and understanding coercive control; (2) barriers to communicating and reporting; and (3) training needs.

### Defining and understanding coercive control

Professionals reflected on their perceptions of parents and young people having *insufficient understanding* of coercive control, and how *healthy and unhealthy relationship modelling* influences the level of understanding of coercive control present within these populations. Most professionals identified that both parents and young people appeared to have difficulties in recognizing coercive controlling behaviours, particularly within the context of their own relationships. This was often attributed to the subtlety of coercive control and ways in which these behaviours can be ‘*masked’* [Professional 4].

‘*I have encountered situations where people aren't maybe aware that that's the situation they are in, both young people and older women …. Pretty common, I would say, in the work that we do, particularly with females, yeah.’* [Professional 2]

Two professionals described how many victims might not become aware that they are/were experiencing coercive control ‘*until they have had a conversation with someone else,’* [Professional 2]. It was also suggested that young perpetrators might similarly be unaware that their behaviour is coercive controlling/abusive.

Some professionals felt that parents and young people may alternatively be aware that the behaviour is inappropriate, but unaware of the specific language used to label this, indicating a disconnection between the behaviour(s) and terminology:

‘*I think it's the use of language as well and how terms are phrased. And labels are almost put on something. Whereas if you explain to young people what actually coercive control is, they're like, yeah, I know what you mean.’* [Professional 5]

The importance of using relatable language to explain coercive control to young people initially, prior to educating young people on specific terminology, was emphasized:

‘*Are you being manipulated? Are you being used? Those types of… that type of language, which is relatable. And getting young people, as part of the training, to start learning some of those words. What is domestic violence? What is domestic abuse? How do we describe it? Give us some examples.’* [Professional 1]

How relationships are modelled to young people within their personal lives and online was also perceived as shaping young people's perceptions of what behaviour is acceptable within intimate relationships, and influencing their risk of perpetration and victimization. Many professionals described how witnessing unhealthy parental relationship dynamics can shape young people's perceptions of how relationships ‘should’ be and may consequently normalize coercive controlling behaviours:

‘*Whether that be a young woman who is controlled by a partner and has maybe seen their own parents, and how their own parents' relationship, so they don't see anything wrong in that. Or from a young man's perspective, who is doing the controlling, maybe that’s the relationship that they have witnessed in their parents.’* [Professional 1]

Some professionals also alluded to an ‘*intergenerational knock down’* [Professional 4] effect, in that certain perceptions, behaviours, and relationship dynamics, which intersect with traditional gender norms and stereotypes, may be passed down throughout families. These perceptions and behaviours were regarded as being deeply entrenched within wider family systems and therefore re-enacted by young people, perhaps unknowingly. As such, the need for education and intervention aimed at families, as opposed to young people in isolation, was highlighted.

The influence of social media on young people's perceptions of how a relationship ‘should’ be, and what this might entail, was also deemed significant by many professionals. Relationship dynamics and content on social media may not only normalize, but also implicitly encourage young people’s acceptance of traditionally unacceptable behaviours, such as cheating. Some professionals suggested that young people might undisputedly model their own relationships based on those they observe on social media, which then serves as justification for certain ‘*toxic*’ behaviours:

‘*I think social media really helps… now, in a negative way helps toxic relationships be sort of… what's the word I'm looking for? Romanticised. … they're looking at these behaviours online and they're like, oh, I wish my partner would get on like that with me. Jealousy and stuff like that.’* [Professional 5].

### Barriers to communicating and reporting

Many professionals identified fear as being a salient emotion associated with the prospect of disclosing coercive control, including disclosures pertaining to witnessing coercive control within the context of parental relationships, and acknowledged that most young people understandably fear the potential negative consequences that might arise from this:

‘*I think one of the examples that would be most common would be the fear of causing more damage at home. Or that they would then potentially be removed from the house. Or that there would be more violence as a result.’* [Professional 2]

Yet fear pertaining to disclosure did not appear to be exclusive to young people and, instead, appeared to be a mutual experience of both young people and professionals:

‘*I think sometimes, when you're put on the spot, you panic yourself, and you're like, right, I could say the wrong thing here. I could actually make it worse…’* [Professional 5]

Moreover, the establishment of trust was overwhelmingly perceived as being a crucial component in facilitating young people to disclose experiences of abuse and/or coercive control. Most professionals identified that young people are highly selective of who they disclose such experiences to, and to what extent:

‘*I know that there’s been situations where there has been young people within a school setting and they have waited and come to us. And whenever the teachers are aware, it’s like, but we had them. We had the conversations and there was nothing. And it is, it’s down to trusting.’* [Professional 2]

The extent of experiences of abuse and/or coercive control disclosed to professionals may remain somewhat limited. It was therefore acknowledged that young people are more likely to both disclose and discuss these experiences more openly with friends or siblings.

Issues regarding the availability of services to support those affected by coercive control were also raised, in addition to a lack of awareness of services among parents, young people and, in some instances, professionals. There was particular uncertainty regarding what services are available at a given time, for specific age groups and/or demographics, such as those under eighteen years old, LGBTQ+ individuals etc.

‘*Just because, sometimes, previously whenever we have phoned services, they have maybe not had funding renewed or they are not working within this section of young people or ages.’* [Professional 2]

This often resulted in sole reliance on outward referral to organizations such as social services and the police. However, one professional alluded to a revolving door effect due to budget cuts and organizational pressures within these organizations, resulting in young people typically falling ‘*back at the feet’* [Professional 3] of the community sector.

Professionals also indicated that *coercive control is a sensitive topic* for a number of reasons. It was felt that both parents and professionals may attempt to distance themselves from the role of educating, and supporting young people around not only coercive control, but also intimate relationships more broadly:

‘*…we have parents that will come and say, I need you to have a conversation. ….. And the parents kind of push the problem on. Now there's two sides. Simply because they panic and they don't know what to do. And the other side is, they don't have the skills either, because they have been out of education or they are not aware of what to say*.’ [Professional 2]

Schools, including teachers, were similarly perceived as avoidant of educating and supporting young people around intimate relationships. Whilst some professionals acknowledged that teachers, like parents, do not necessarily possess the skillset required to educate young people on relationships in a ‘*relatable’* manner [Professional 1], there was an overall sense that sex education and coercive control remain ‘*taboo’* [Professional 3] topics. It was therefore implied that there is a wider societal attitude whereby ‘*you don’t talk about that*,’ [Professional 5] which may underlie many parents' and professionals' reluctance to genuinely discuss relationships and topics associated with these, such as coercive control, with young people.

For parents and young people with lived experience of coercive control, it was felt that discussions around this topic may ‘*trigger*’ [Professional 2] them, and/or evoke some resistance:

‘*… it strikes a chord because they're going, what? What do you mean that men shouldn't tell women what to do? Or, I'm in a relationship with a girl and she shouldn't be looking at anybody else or talking to anybody else. Those types of things that they have witnessed, then when you start so speak into them, it challenges them.’* [Professional 1]

Such discussions may have a significant emotional impact on these parents and young people, as their understanding of their own experiences may be questioned and redefined, resulting in shock and/or distress.

## Training needs

Professionals provided direction on the practical considerations associated with developing training for parents, professionals and young people, including the content and delivery methods used. It was felt that some supports could be digital (e.g. games and exercises with scenarios; websites; social media content) as ‘*young people are spending more and more time online’* [Professional 2], and while social media has a negative side it can also be used ‘*positively to spread the word of the likes of these things’* [Professional 5]. While there was an overall sense that professionals were conflicted on what they perceived to be the ‘*ideal*’ delivery setting between formal (i.e. schools) and informal settings (i.e. youth clubs), professionals were clear in their view that a variety of stakeholders need to be involved in education delivery and that no single organization or setting should bear this responsibility solely:

‘…*I think you capture a wider audience if you get the schools, because it's compulsory to be there. And then for those young people that are attached to the youth services, then you can go deeper and further with it*.’ [Professional 7]

Tailoring training delivery to ensure inclusivity and meaningful engagement for all young people was a prominent point of discussion. Again, there appeared to be a sense of uncertainty regarding how to implement this, as professionals vacillated between the potential for single-demographic (e.g. all male, all LGBTQ) and mixed cohorts.

‘*particularly if young men are on the receiving end of domestic violence, you know, and they're not going to speak about it in the context of an all-male gender grouping. Or is it better having mixed gender?’* [Professional 1].

One professional pointed out the increased heterogeneity of Northern Ireland's population, therefore indicating a demonstrable need to diversify content delivered through training to reflect this:

‘*We need to consider a wide range of relationships. …we don't just have heterosexual and gay and bisexual relationships any longer. There's a raft. And I think people need to have that information.’* [Professional 3]

‘*Because it's not about targeting refugee and asylum groups. It's about targeting everybody and making sure refugee and asylum groups are included within that*.’ [Professional 4]

It was apparent that professionals believed training delivery would need to be versatile, rather than adopting a ‘one-size fits all’ approach and offered within a ‘safe space’ for young people to encourage meaningful engagement across all populations.

Additionally, professionals shared perspectives on the specific training needs of parents, professionals and young people. Increasing parents' awareness of and knowledge pertaining to coercive control was emphasized in discussions regarding parental training needs:

‘*So I think what we would need to put a message out is, this is how subtle this can happen and how it can affect your child. If it's happening in your home, this is how it can impact long term for your child*.’ [Professional 1]

It was felt that facilitating parents' ability to protect their children from coercive control would be an invaluable component of parental training. Most professionals therefore felt that parental training should aim to equip parents with the skills to confidently approach and navigate such conversations, in addition to the knowledge required to educate young people on coercive control.

Similarly, how to identify/recognize coercive control (e.g. what behaviours does it entail) was considered to be a key training need for professionals. Participants also expressed a need for increased awareness amongst professionals regarding how coercive control may present differentially within various contexts and among specific populations, such as the LGBTQ+ community. Beyond this, managing disclosures effectively, and generally ‘*knowing what to say’* when discussing healthy and unhealthy relationships with young people, [Professional 5], seemed to be focal areas in which professionals expressed training needs, particularly when working with young men.

‘*What's our role? What's our responsibility? And how do we look at this, how do we tackle this?’* [Professional 1]

‘*I'm a big believer of, it's not just males who are the issue. And it has become more and more apparent that there actually is females that are being the perpetrators and being these individuals too. But we are struggling with males. … But it's trying to get males together. And there is something there around how males speak, the language then, that is fed down to their young people, that I think is detrimental to how sometimes young males particularly then behave. It's like learned behaviour’ [Professional 2]*

It appeared that a lack of clear guidance regarding potential next-steps following onward referral to the police and social services etc., evoked disappointment and perhaps frustration, and it seemed that professionals hoped training might remedy this by clarifying the scope of their role.

Again, increasing awareness of and knowledge pertaining to coercive control, in addition to where to seek relevant support, was discussed in relation to young people's training needs. However, professionals ultimately highlighted that the development of broader life skills, should be a focal component of training for young people:

‘*It's just teaching young people resilience as well, around relationships and knowing what’s OK, what's not OK and how to deal with that situation.’* [Professional 5]

‘…*if you can do conflict well, if you can do conflict resolution, if you can know when to apologise but also stand firm and wait for an apology.’* [Professional 7]

Professionals indicated that training for young people should encourage self-discovery and skill development that is generalizable to wider relationship contexts. Many felt training should first encourage young people to build confidence in themselves and how they relate to others so that they can then apply associated learning on what is healthy and unhealthy in relationships.

## Discussion

The present study sought to capture the views of youth work professionals to facilitate the development of HYAR education resources for young people that could be delivered through the youth work sector. The first master theme, ‘*defining and understanding coercive control*,’ outlined what professionals perceived to be deficits in parents' and young people's understanding of coercive control, which were characterized by difficulties in both recognizing coercive controlling behaviours and articulating experiences of this. The second master theme, ‘*barriers to communicating and reporting*,’ highlighted various factors that were perceived to negatively influence communication and reporting of coercive control. These factors included barriers to help-seeking, such as fear and trust (or lack thereof), gaps in availability and awareness of support services, and coercive control being perceived as a sensitive topic due to individuals' lived experiences of this and wider societal attitudes. Finally, key findings from the third master theme, ‘*training needs*,’ provided insight into professionals' perspectives on the multifaceted nature of training delivery and the specific training needs of parents, professionals and young people. Indeed, a whole-community approach to HYAR education delivery was strongly endorsed by professionals, with specific emphasis being placed on the necessity for shared responsibility. It was also acknowledged that young people require a safe space to meaningfully engage in HYAR education and that training should be tailored to ensure inclusivity and reflect a diversity of relationships. With regard to training, there was consensus that parents, professionals and young people should be educated on coercive control, yet it was simultaneously agreed that this training should seek to facilitate broader life skills development, particularly for young people.

According to professionals within the current study, both parents and young people appeared to struggle with not only recognizing coercive control, but also with the specific terminology used to label and describe this pattern of behaviour. These findings are consistent with previous research (Abbott et al., 2021; Martin et al., 2012), including that of Lagdon et al. (2023), in which only 62.9% of adults and 16% of young people in NI had heard of the term coercive control or knew what this meant. Future work could include collecting data to benchmark this finding, e.g. how many people know what gaslighting means? Similar findings relating to a perceived gap in awareness among young people have also been identified in research involving professionals in formal education, health and municipal social services in other regions, such as Spain (Maquibar et al., 2017). Moreover, professionals within the present study emphasized the role of observational learning on young people's level of understanding regarding relationships and, by extension, coercive control. Young people themselves have similarly identified observation of relationship dynamics portrayed by family, friends and media as being a method of learning about what an ideal relationship might look like (Kedzior et al., 2024), particularly for those with limited relationship experience (Taba et al., 2020). As such, it is apparent that young people derive their understanding of relationships, including what behaviour is (in)appropriate within relationships, from multiple, complex relationship models that both influence and are influenced by existent relationship norms and attitudes. This is an important area for addressing within future HYAR education. Indeed, the actualities of ‘real’ relationships and inclusion of ‘relatable content’ have previously been identified by young people (Setty, 2023).

Further, various factors, including fear, trust (or lack thereof) and perceptions of coercive control as being a sensitive topic, were identified by professionals within the current sample as presenting a barrier to communicating and reporting experiences of coercive control, particularly between young people and adults. In previous research (e.g. Martin et al., 2012), fear of further violence and shame have been cited by young people as preventing them from reaching out for support when faced with relationship violence; the latter of which may be particularly salient in the context of the current findings. The professionals in the present study felt that, due to trust issues, young people may be more likely to disclose such experiences to peers or siblings. Somewhat similar findings have been evidenced in research conducted among young people, in which young people, especially young males, have disclosed a preference for initially turning to friends, older siblings and family members, rather than seeking formal support (Martin et al., 2012). Yet, existing literature indicates that informal supports, such as friends and family members, may inadvertently fail to manage disclosures sensitively or effectively, through minimizing the severity of the abuse, not offering help or blaming the victim (Ahrens et al., 2007; Fanslow & Robinson, 2010). Professionals in this study felt that parents were reluctant to discuss sensitive relationship topics with their children, and previous research suggests that this likely stems from lack of knowledge, low self-efficacy, and concerns about encouraging risk behaviours (Jaccard et al., 2002); this could potentially be addressed via structured psychoeducation and communication skills training (e.g. non-judgemental responding, active listening, example prompts) that builds parental confidence and increases the frequency of relationship-focused discussion (e.g. Akers et al. 2011; Widman et al., 2016).

### Implications

The current research findings present a number of implications for both policy and practice. Firstly, there is a clear implication for perspectives regarding who is deemed responsible for the delivery of educational programmes concerning coercive control and unhealthy relationships. Whilst increased emphasis has been placed on the role of formal educators in the delivery of RSE (e.g. Cumper et al., 2024; Cole, 2018, Martínez et al., 2012), the concerns that RSE has now been tasked with addressing, such as coercive control, have simultaneously grown in complexity (Stevens, 2023). Additionally, as the present results show, the establishment of multiple informal sources of support and guidance will maximize the likelihood that young people with trust issues in authority figures will feel they have someone they can disclose to. Finally, as the professionals highlighted, those who grow up in households where unhealthy relationships are modelled must have the opportunity to learn about HYAR pre- and post-16 years. Therefore, the broader social environments within which individuals are embedded (e.g. families, peers, communities, institutions) should be involved in prevention-practice initiatives. In light of the present findings, there is a clear recommendation that a whole-community approach to HYAR education, encompassing both formal (e.g. schools) and informal settings (e.g. youth work sector, families), should be adopted, and that education delivery should be inclusive, versatile and offered within a ‘safe space,’ to ensure meaningful engagement across all young people. This may include approaches such as Restorative Practice (McCluskey et al. 2008) and Trauma-Informed Care (Bunting et al. 2019) being integrated into youth worker training to support staff when tasked with building trust and managing disclosures in a sensitive manner. Further, given the lack of opportunities for referral and support systems in place highlighted by youth workers, services need to develop and implement clear referral pathways to local support services for children and young people affected by abuse as per National Institute for Health and Care Excellence recommendations (NICE, 2014). Our results suggest particular uncertainty amongst professionals regarding available provision for under 18 s and those from the LGBTQ community, hence clear pathways to support should be developed to prevent any gaps in provision.

Undeniably, youth work professionals are often at the forefront of attempting to address young people's relationship issues (Zero Tolerance & YWCA Scotland, 2012), yet those employed within this sector appear to be provided with little specialist professional development and guidance to facilitate this. As such, these findings query the suitability of existing support frameworks for youth workers, particularly given the increasing expectations regarding relationship dynamics without formalized professional development and training. A clearer separation between pastoral care, education, and safeguarding tasks is also needed to prevent burnout and ensure that the scope of practice reflects resources available.

The participants conveyed a willingness to support young people's HYAR, but in line with Walker et al. (2021), emphasized the need to enhance current training provision to build their confidence in the subject matter and their capacity to educate/signpost young people and their parents. The professionals recommendations were threefold: (1) the creation of training/educational materials for youth work professionals that will give them the skills and confidence they need to support young people involved in unhealthy relationships; (2) development of interventions that can be delivered through the youth work sector to young people; (3) materials/resources aimed at parents that youth workers can signpost parents to as appropriate. In relation to the former, the professionals were very aware of their personal educational needs and highlighted the need for resources, training and guidance focused on how to recognize coercive control in a variety of contexts (e.g. LGBTQ relationships), clarity around their safe guarding role, how to manage disclosures including knowing what to say to the young person, and suitable referral routes. Formal supervision, as seen within other professions, such as social work and clinical psychology, could play a useful role in supporting youth work professionals who are dealing with tricky disclosures by providing a dedicated space to talk through challenges, as well as receive emotional and practical support. The findings also suggest a need for clearer guidance for staff responding to disclosures of coercive control. Practitioners operate in contexts where the boundaries between informal support, therapeutic engagement and safeguarding can often be blurred. More formalized training could support decision-making and achieve a balance between formal legal and professional obligations with informal support. These resources are likely needed by other professionals as previous research has shown formal educators, such as teachers, expressed the need for more appropriate support, experience and subject knowledge around issues relevant to RSE (e.g. Cumper et al., 2024).

With regard to training needs of young people and their parents, many professionals indicated a perceived need for education and training relating to coercive control (e.g. how to recognize it; key terminology) and broader skill-building among parents and young people. One of the implications in the participant data was that youth workers are gradually being asked to take on responsibilities that extend into therapeutic territory. Unlike clinical colleagues, however, they often don't receive the training or supervision that such work would normally require. As such, real concerns arise about how sustainable these expectations are, and the emotional toll it may take as well—particularly if professional boundaries are becoming blurred in the absence of clearer guidance.

Several professionals highlighted a role for digital supports in educating young people about coercive control. Whilst a purely digital solution may not be the way forward, such approaches may form part of a wider range of measures to educate young people about healthy and unhealthy relationships, including coercive control. A benefit of digital health interventions is that they can be delivered at scale to the population at relatively low marginal cost, easily adapted to different age groups and cultural contexts, and feel less judgemental and more private than face-to-face contact; albeit ‘digital exclusion’ is a risk. However, the evidence base in this area is currently very limited. Indeed, a scoping review of digital interventions (e.g. video vignettes and readings; game interventions) to promote healthy romantic relationships in adolescents revealed only small improvements in romantic relationship communication skills, and concluded that further research with validated measures is needed (Emerson et al., 2023). A particularly notable gap in the evidence base is digital interventions which aim to educate young people about coercive control. Given that the present research suggests that young people struggle to identify coercive control, such interventions should include a wide range of videos and vignettes to reflect the variable nature of coercive control. Further, personalization should also be available to allow the content to be tailored to the gender, sexual orientation and cultural background of the user. For digital interventions aimed at youth workers, allowing them to tailor examples around who they are working with would be useful as in the present research they were unsure about how coercive control manifests LGBTQ relationships. These resources could also include example discussion prompts and strategies to use with different populations (e.g. young males).

### Limitations

A number of limitations should be considered when interpreting the present findings. Firstly, as the current study focuses on the perspectives of those employed within the youth work sector, these findings may not be transferable to professionals employed in other sectors, such as those in formal education (i.e. teachers). Likewise, it is important to reflect on the fact that a different set of interview respondents within the youth sector may have formulated a different opinion; this is a persistent limitation of smaller samples in qualitative research. Whilst professionals within the current sample have shared their views relating to formal education settings in the context of HYAR education and training, future research should also seek to capture the perspectives of those employed within such settings. Additionally, as the present sample consisted primarily of women, greater diversity in relation to sample characteristics may offer further insight into youth work professionals' understanding of coercive control and how to report, monitor, and respond to IPV within young people's relationships. It is also pertinent to reflect upon the fact that we did not query anything about the youth workers' own histories of trauma, or indeed IPV/coercive control in their own intimate relationships and so we have to be conscious that their perceptions may be impacted by their own experiences. We also acknowledge that the use of interviews and organization-based focus groups, designed to accommodate multiple professionals within a single organization, may have introduced certain limitations. In particular, this format carries the potential risk of ‘groupthink,’ whereby consensus-seeking dynamics may limit the expression of divergent views. However, our observations suggest that the focus group format facilitated richer contextual insights and fostered a collegial environment conducive to open discussion. Nevertheless, future research should consider balancing this approach with alternative methods to mitigate potential group dynamic effects and to ensure a breadth of perspectives. A final limitation relates to our results being specific to the sociopolitical and cultural context of NI and professionals who work here. Arguably, we may face similar issues across other nations, but we also have some nuanced differences related to the legacy of political violence; therefore, we must exercise caution in generalizing these results from within NI to the other nations.

## Conclusion

For young people, experiences of forming and navigating romantic relationships can serve as a distinct pathway for future relationship success and personal wellbeing (Exner-Cortens et al., 2013). Given that young people may unfortunately experience coercive control during this process (Barter, 2009), there is a demonstrable need to develop and evaluate early educational interventions targeting this population (Stanley et al., 2015), including those delivered within the youth work sector. As Northern Ireland, like many other regions, continues to lack an evidence-based, standardized RSE curriculum, it is apparent that youth work organizations will continue to assume a key role in the delivery of HYAR education and training. As such, it is intended that the present study, in addition to the wider HYAR research programme, might facilitate the development of one such intervention. Yet, there is consensus that the delivery of HYAR education and training is multifaceted in nature, and the responsibility of this should not fall on a single organization or sector, but rather, consist of a concentrated effort across a variety of stakeholders and settings, including schools and families, to ensure that it is accessible, effective and meaningful for all young people.

## Supplementary Material

Supplementary Material Supplementary Materials.docx

## Data Availability

The participants of this study did not give written consent for their data to be shared publicly, so due to the sensitive nature of the research supporting data is not available.
