# Supplementary material for: Healthy young adult relationships (HYAR): professionals' perspectives on HYAR education and training
Source: Int J Adolesc Youth. 2026 May 8;31(1):2670387. doi: 10.1080/02673843.2026.2670387 (PMC13159938; doi:10.1080/02673843.2026.2670387)
Supplement: Supplementary Material — s.docx [file RADY_A_2670387_SM3666.docx]

***Professional Interview Schedule***

1. **We use a term to describe non-physical abuse in relationships, the term is coercive control. Have you heard of this term?**

- Do you know what it means?
- If yes, were did you hear about this? Was the information useful?

***Researcher will explain:*** Coercive control is described as a pattern of behaviour (so it happens more than one or two times) which aims to intimidate, frighten, control or belittle a person. This might include obsessively texting, calling and emailing isolating them from friends and family, stopping them from working or going to school/college/university; getting upset when they text or hang out with other people; accusing them of flirting or cheating all the time; pressuring them to do things they don't want to; monitoring or controlling their social media accounts; tracking their location via GPS; rushing the relationship’s pace or saying things like “If you loved me you would…”.

- Do you think this happens in young adult relationships?
- Do you think this behaviour is wrong?
- Do you think this behaviour is domestic abuse?

1. **Have you received any training with regards to domestic abuse within young adult relationships and how to respond to this?**

- If yes, who provided this? Did you find it useful?
- If no, is this something you would like to have in the future? What types of information should be included?
- What would be the best mode of delivery?

1. **Has your organisation provided young people with information about healthy relationships?**

- Was this a programme? If yes, who provided the organisation with this?
- Do you think this information was good? What was good about it?
- If you did not find that the information for young people was helpful, why was this?
- Did this programme of information talk about unhealthy relationships?

1. **Do you think young people should be provided with more information about healthy and unhealthy relationships?**

- What types of things should be included in the information programme?
- Who should be delivering the programme for example Teachers? Youth workers? Older Peers?

1. **In what ways should we be making young people aware of healthy and unhealthy relationships?**

- Should this be through an online course? In persons or maybe both?
- What about social media?
- Should information also be included on posters or maybe TV ads?
- Do you think schools /youth groups should be providing this information?
- Who else should be advising on this information?

1. **Should parents or guardians be provided with information about healthy and unhealthy young adult relationships?**

- What types of things should they know?
- What types of things might help parents or guardians improve how they discuss healthy and unhealthy relationships with young people?

1. **If a young person was in an unhealthy relationship, who do you think they would likely tell?**

- **Do you think they could speak with a teacher or youth worker?**
- **Would they prefer to tell a friend?**
- **Would they tell the police?**

**What do you think the young person would want to happen next if they told someone?**

1. **Are you aware of any support services for young people who experience coercive control?**

- What about services for domestic violence and abuse?
- Do you think if they had to, a young person would use these services? If not, why not?

1. **What further help and support is needed for young people who might be in an unhealthy or abusive relationship?**
2. **Is there anything further that could be done to raise awareness/increase understanding of coercive control and domestic abuse more broadly among young people?**
